# Allocation of the S-genome chromosomes of *Aegilops variabilis* Eig. carrying powdery mildew resistance in triticale (× *Triticosecale* Wittmack)

**DOI:** 10.1007/s00709-015-0813-6

**Published:** 2015-04-14

**Authors:** M. Kwiatek, J. Belter, M. Majka, H. Wiśniewska

**Affiliations:** Institute of Plant Genetics, Polish Academy of Sciences, Strzeszyńska 34, 60-479 Poznań, Poland

**Keywords:** *Aegilops*, Chromosome transfer, In situ hybridization, Molecular marker, Powdery mildew, Resistance genes, Triticale

## Abstract

It has been hypothesized that the powdery mildew adult plant resistance (APR) controlled by the *Pm13* gene in *Aegilops longissima* Schweinf. & Muschl. (S^l^S^l^) has been evolutionary transferred to *Aegilops variabilis* Eig. (UUSS). The molecular marker analysis and the visual evaluation of powdery mildew symptoms in *Ae. variabilis* and the *Ae. variabilis* × *Secale cereale* amphiploid forms (2*n* = 6*x* = 42, UUSSRR) showed the presence of product that corresponded to *Pm13* marker and the lower infection level compared to susceptible model, respectively. This study also describes the transfer of *Ae. variabilis* Eig. (2*n* = 4*x* = 28, U^v^U^v^S^v^S^v^) chromosomes, carrying powdery mildew resistance, into triticale (× *Triticosecale* Wittm., 2*n* = 6*x* = 42, AABBRR) using *Ae. variabilis* × *S. cereale* amphiploid forms. The individual chromosomes of *Ae. variabilis,* triticale ‘Lamberto’ and hybrids were characterized by genomic and fluorescence in situ hybridization (GISH/FISH). The chromosome configurations of obtained hybrid forms were studied at first metaphase of meiosis of pollen mother cells (PMCs) using GISH. The statistical analysis showed that the way of S-genome chromosome pairing and transmission to subsequent hybrid generations was diploid-like and had no influence on chromosome pairing of triticale chromosomes. The cytogenetic study of hybrid forms were supported by the marker-assisted selection using *Pm13* marker and visual evaluation of natural infection by *Blumeria graminis*, that allowed to select the addition or substitution lines of hybrids carrying chromosome 3S^v^ which were tolerant to the powdery mildew infection.

## Introduction

Powdery mildew caused by *Blumeria graminis* (DC.) E.O. Speer f. sp*. Tritici* Em. Marchal *(Bgt) = Erysiphe graminis* DC. Ex Merat f. sp. *Tritici* Em. Marchal is one of the widespread fungal diseases in cereals. This pathogen has recently infected triticale (× *Triticosecale* Wittm.), man-made, artificial cereal, which was created to combine the characteristics of cold, disease tolerance and adaptation to unfavourable soils and climates with the productivity and nutritional qualities (Woś et al. 2002). At the beginning of the triticale production, the diseases did not appear to be a serious limitation, probably because of lack of the appropriate, triticale-directed pathotypes of fungal pathogens. Moreover, the grown areas of this crop were incidental to cause serious shifts in the pathogen virulence (Ammar et al. 2004). While the harvest area of triticale began to increase, the new hybrid pathotypes carrying virulence genes appeared (Arseniuk 1996). The new, resistant cultivars could eliminate the fungicides accumulation in grain and reduce the crop losses caused by powdery mildew. Two types of resistance to powdery mildew have been identified so far (Flor 1971). First is called monogenic (vertical) or rac-specific resistance, which is effective for some isolates of the pathogen, but ineffective for others. Race-specific resistance is expressed in seedlings and involve single major R genes, in a gene-for-gene interaction (Chen and Chełkowski 1999). Race-specific resistance genes are widely used to combat the wheat diseases, yet the resistance is often short-lived, especially when the genes are employed singly in new varieties (Marais et al. 2008). Second type of resistance to powdery mildew is known as an adult plant resistance (APR), also called ‘slow mildewing’ and ‘partial resistance,’ which decelerates the infection, growth and reproduction of the pathogen in adult plants. APR to powdery mildew is more durable than race-specific resistance; therefore it is more desirable in breeding programmes. One of the APR genes is *Pml3* powdery mildew resistance gene that ensures high tolerance to all known races of this disease in wheat. The *Pm13* gene has been transferred from the chromosome 3S^1^ of *Aegilops longissima* Schweinf. & Muschl. (2*n* = 2*x* = 14 chromosomes; S^l^S^l^) into common wheat, *Triticum aestivum* L. cv. ‘Chinese Spring’ (Ceoloni et al. 1988). Considering the synteny in the genome construction of related species, which evolved from a common ancestral gene by speciation, Cenci et al. (2003) hypothesized that the *Pm13* marker linked with powdery resistant gene has a conservative character. On this basis, it can be assumed that species with S-genome chromatin such as tetraploids (*Aegilops variabilis* Eig.) and hexaploids (*Aegilops vavilovi* Zhuk.) could carry the genomic region responsible for powdery mildew resistance. What is more, *Ae. longissima* is considered as a donor of S-genome (Yu and Jahier 1992; Zhang et al. 1992; Badaeva et al. 1998) of *Ae. variabilis* (U^v^U^v^S^v^S^v^). *Ae. variabilis* has been used as a donor of desirable genes to wheat through interspecific hybridization such as powdery mildew resistance (Spetsov et al. 1997), leaf rust resistance (Marais et al. 2008) and resistance to nematodes (Coriton et al. 2009).

The aims of this study were to: (1) evaluate the presence and the expression of *Pm13* gene in *Ae. variabilis*; (2) to identify the individual chromosomes of *Ae. variabilis* responsible for powdery mildew resistance and (3) transfer them into triticale.

The distant crossing between diploid *Aegilops* species and hexaploid triticale can be disturbed because of (1) different ploidy level of the parental components and (2) the expression of *Ph1* gene located on chromosome 5B in wheat (or triticale), responsible for homologues chromosome pairing during meiosis (Riley and Chapman 1958; Lukaszewski and Kopecký 2010). To avoid the unwanted crossing limitations connected with different chromosome number in parental forms and to circumvent the chromosome pairing system controlled by *Ph1* gene, we assumed that using amphiploid forms of *Ae. variabilis* × *Secale cereale* (U^v^U^v^S^v^S^v^RR) in the crosses with triticale (AABBRR) will have a significant impact on F_1_ hybrid stability because of R-genome chromosomes, which will be able to pair during prophase I of meiosis and will ensure the functional daughter cells formation and sufficient level of vital pollen grains as a consequence.

In this purpose, four subsequent generations (F_1_ to BC_2_F_2_) of (*Ae. variabilis* × *S. cereale*) × triticale hybrids were obtained. The chromosome composition during metaphase of mitosis in root apical meristems and chromosome pairing during metaphase I (MI) of meiosis of the pollen mother cells (PMCs) were characterized using fluorescence and genomic in situ hybridization (FISH/GISH). Finally, the *Pm13* marker (Cenci et al. 1998) was verified in the *Ae. variabilis*, parental components and in the hybrid plants and compared with visual evaluation of powdery mildew infection.

## Materials and methods

### Plant material

Glasshouse experiments were carried out in four subsequent vegetation seasons at Institute of Plant Genetics, Polish Academy of Sciences in Poznań, Poland. Seeds of *Aegilops umbellulata* Zhuk. (PI 222762; 2*n* = 2*x* = 14; U^u^U^u^) and *Ae. longissima* (PI 604112; 2*n* = 2*x* = 14; S^l^S^l^) were kindly supplied for the study from the National Small Grains Germplasm Research Facility, National Small Grains Collection (Aberdeen, Idaho, USA). Seeds of *Ae. variabilis* were received from the collection of Professor M. Feldman (The Weizmann Institute of Science, Israel). The *Ae. variabilis* × *S. cereale* amphiploids (U^v^U^v^S^v^S^v^RR, 2*n* = 6*x* = 42) were obtained by Wojciechowska and Pudelska (1999). The F_1_ (*Ae. variabilis* × *S. cereale*) × triticale hybrids were obtained by crossing of triticale cv. ‘Lamberto’ with *Ae. variabilis* × *S. cereale* amphiploids as a pollinator. Backcrosses with the triticale as a male parent were used to achieve following generations (BC_1_F_1_ and BC_2_F_1_). Finally, the self-pollinations of BC_2_F_1_ hybrids were made to gain BC_2_F_2_ plants. The percentage ratio of the total amount of seeds from each plant with the total amount of pollinated flowers of each plant was calculated (Table 1).

**Table 1:** Results of distant crossing between hexaploid (2*n* = 6x = 42) forms of triticale ‘Lamberto’ with *Ae. variabilis* × *S. cereale* amphiploid and its progeny

Hybrid generation	Cross combination		Number of pollinated flowers	Number of seeds obtained	Crossability	Number of adult plants with *Pm13* marker
	Female parent	Male parent				
F_1_	triticale (*6x*)	*Ae. variabilis* × *S. cereale* (*6x*)	106	19	0.18	6
BC_1_F_1_	F_1_	Triticale (*6x*)	68	17	0.25	5
BC_2_F_1_	1	Triticale (*6x*)	46	3	0.07	0
	3	Triticale (*6x*)	116	6	0.05	0
	4	Triticale (*6x*)	82	11	0.13	11
	6	Triticale (*6x*)	30	2	0.03	1
	7	Triticale (*6x*)	56	3	0.05	3
BC_2_F_2_	4/1	Self	64	0	0	0
	4/2	Self	44	0	0	0
	4/3	Self	52	0	0	0
	4/4	Self	48	0	0	0
	4/5	Self	74	27	0.36	2
	4/6	Self	64	3	0.05	3
	4/7	Self	60	0	0	0
	4/8	Self	40	0	0	0
	4/9	Self	52	0	0	0
	4/10	Self	73	10	0.13	10
	4/11	Self	66	10	0.15	10
	6/1	Self	68	0	0	0
	7/1	Self	48	0	0	0
	7/2	Self	17	0	0	0
	7/3	Self	52	0	0	0

### Chromosome preparation

Seeds were germinated on moist filter paper in Petri dishes for 3–4 days. For mitosis metaphase accumulation, the root-tips were collected and stored in ice for 26 h. Afterwards, the plants were placed in the vernalisation chamber for 6 weeks and then located in the glasshouse until harvest. The fixation of the root-tips was made using ethanol and acetic acid (3:1, *v*/*v*). The chromosome preparations were made according to Hasterok et al. (2006). The F_1_ to BC_2_F_2_ hybrids were grown in the nursery and their meiotic behaviour was analysed in PMCs at MI of meiosis. Anthers of the hybrids containing PMCs at MI were fixed in 1:3 (*v*/*v*) acetic acid/ethanol and stored at −20 °C for a maximum of 2 months. MI of meiosis preparations were made according to Zwierzykowski et al. (2008). The anthers were squashed in 45 % acetic acid, and the slides were stored at 4 °C until in situ hybridization.

### Probe labelling

Total genomic DNA was extracted from fresh leaves of *Ae. umbellulata* (UU), *Ae. longissima* (S^l^S^l^) and triticale ‘Lamberto’ (AABBRR) using GeneMATRIX Plant & Funghi DNA Purification Kit (EURx Ltd.). Genomic DNA from *Ae. umbellulata* and *Ae. longissima* was labelled by nick translation (using NickTranslation Kit, Roche, Mannheim, Germany) with digoxigenin-11-dUTP (Roche) or tetramethyl-5-dUTP-rhodamine (Roche), respectively. Blocking DNA from triticale was sheared to fragments of 5–10 kb by boiling for 30–45 min and used at a ratio of 1:50 (probe:block). The 5S rDNA probe was amplified from the wheat clone pTa794 (Gerlach and Dyer 1980) by polymerase chain reaction (PCR) with tetramethyl-rhodamine-5-dUTP (Roche) using universal M13 ‘forward’ (5′-CAG GGT TTT CCC AGT CAC GA-3′) and ‘reverse’ (5′-CGG ATA ACA ATT TCA CAC AGG A-3′) sequencing primers. The thermal cycling programme consist of the following: 94 °C for 1 min, 39 cycles of 94 °C for 40 s, 55 °C for 40 s, and 72 °C for 90 s, and 72 °C for 5 min. The 25S rDNA probe was made by nick translation of a 2.3-kb *Cla*I sub-clone of the 25-5.8-18S rDNA coding region of *Arabidopsis thaliana* (Unfried and Gruendler 1990) with digoxigenin-11-dUTP (Roche). It was used for detection of 25-5.8-18S rDNA loci. The pSc119.2 repetitive DNA sequence, kindly supplied from Dr Kubalaková (Laboratory of Molecular Cytogenetics and Cytometry, Institute of Experimental Botany, Olomouc, Czech Republic), was amplified and labelled by PCR with digoxigenin-11-dUTP (Roche) by using universal M13 primers (Vrána et al. 2000). The probe pAs1 (*Afa* family) was amplified by PCR from the genomic DNA of *Ae. tauschii* and labelled with digoxigenin-11-dUTP (Roche) according to Nagaki et al. (1995). Digoxigenin detection was made using anti-digoxigenin-fluorescein antibody (Roche).

### In situ hybridization

FISH was carried out to study the mitotic chromosomes of root meristems. On the other hand, GISH was used to examine both the mitotic chromosomes of root meristemes and meiotic chromosomes of PMCs. Four probes were subjected to in situ hybridization on the same chromosome preparations. First FISH was made according to Książczyk et al. (2011) with minor modifications of Kwiatek et al. (2013), using 25S (used for detection of 25-5.8-18S rDNA *loci*) and 5S rDNA (pTa794). The hybridization mixture (40 μl per slide) contained 90 ng of each probe in the presence of salmon sperm DNA, 50 % formamide, 2 × SSC, 10 % dextran sulphate, and was denatured at 75 °C for 10 min and stored on ice for 10 min. Chromosomal DNA was denatured in the presence of the hybridization mixture at 75 °C for 5 min and allowed to hybridize overnight at 37 °C. For detection of the hybridization signals, anti-digoxigenin conjugated with FITC (Roche) was used. After documentation of the FISH sites, the slides were washed according to Heslop-Harrison (2000) (2 × 45 min in 4 × SSC Tween, 2 × 5 min in 2 × SSC, at room temperature).

Second FISH with pSc119.2 and pAs1 (labelled with digoxygenin-11-dUTP and tetramethyl-rhodamine-5-dUTP, respectively) was made with the same conditions after reprobing. After second reprobing, GISH was carried out according to Kwiatek et al. (2012) with modifications. Multicolour GISH was carried out using U-genome probe (from *Ae. umbellulata*), S^l^-genome probe (from *Ae. longissima*) and unlabelled triticale genomic DNA which was used as specific blocker. The GISH mixture (40 μL per slide), containing 50 % formamide, 2 × SSC, 10 % dextran sulphate, 90 ng each of the genome probes, and 4.5 μg blocking DNA, was denatured at 75 °C for 10 min and stored on ice for 10 min. In case of initial GISH on triticale ‘Lamberto’ chromosomes, the hybridization mix contained the following: A-genome probe generated from genomic DNA of *Triticum monococcum* L., R-genome probe (rye, *S. cereale* L.) and blocking DNA from B-genome (*Aegilops speltoides* Tausch; 2*n* = 2*x* = 14; SS). The chromosomal DNA denaturation, hybridization and immunodetection conditions were the same as above-mentioned. Mitotic and meiotic (MI) cells were examined with an Olympus XM10 CCD camera attached to an Olympus BX 61 automatic epifluorescence microscope. Image processing was carried out using Olympus Cell-F (version 3.1; Olympus Soft Imaging Solutions GmbH: Münster, Germany) imaging software and PaintShop Pro X5 software (version 15.0.0.183; Corel Corporation, Ottawa, Canada). The identification of particular chromosomes were made by comparing the signal pattern of 5S rDNA, 25S rDNA, pSc119.2 and pAs1 probes according previous study (Kwiatek et al. 2013) and similar cytogenetic analysis (Cuadrado and Jouve 1994; Schneider et al. 2003, 2005; Wiśniewska et al. 2013). Single-factor analysis of variance and Tukey’s Honest Significant Difference (HSD) test was used to examine the differences of means of chromosome configurations between plants from respective generations and the differences of means of chromosome configurations between plants from BC_2_F_1_ with comparison to their progeny in BC_2_F_2_ generation.

### PCR amplification of powdery mildew resistance gene marker

Genomic DNA was extracted from fresh leaves of single plants using GeneMATRIX Plant & Funghi DNA Purification Kit (EURx Ltd.). Total genomic DNAs of F_1_ to BC_2_F_2_ hybrids were used as templates for PCR. The reaction was performed in 25 μl reaction mixture containing: 1.5 μl 50 ng/μl of DNA, 2.5 μl 10 × PCR buffer (50 mM KCl, 1.5 mM MgCl_2_, 10 mM Tris-HCl, pH 8.8, 0.1 % Triton X-100), 1 μl 2.5 mM dNTPs (Thermo Fisher Scientific, Waltham, MA, USA), 12.5 pmol of each primer (UTV14 forward: CGC CAG CCA ATT ATC TCC ATG A and UTV14 reverse: AGC CAT GCG CGG TGT CAT GTG AA; Cenci et al. 1998) (Sigma), and 16 μl MQ H_2_O, 0.5 μl (2 U/μl) Taq Polymerase (Thermo Fisher Scientific). Amplifications were carried out in LabCycler thermocycler (SensoQuest Biomedizinische Elektronik, Goettingen, Germany). Amplification products were electrophoresed at 5 V/cm for about 3 h in 1.5 % agarose gel (Sigma), stained with ethidium bromide (Sigma), visualized under UV light and photographed (Syngen UV visualiser).

### Evaluation of the powdery mildew infection

**Fig. 1:**
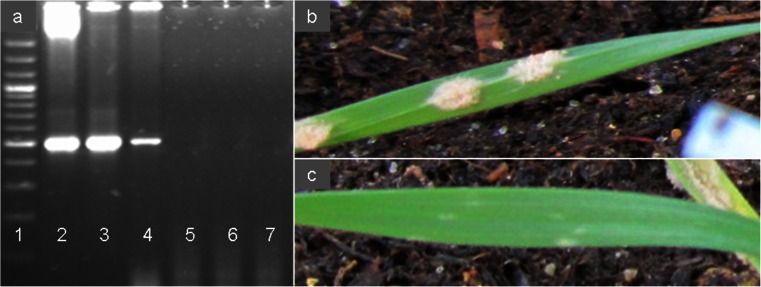
**a** Amplification products (517 bp) of PCR with primers specific to *Pm 13* gene marker. *Lane 1* - 100 bp ladder (GeneRuler, Thermo Fischer Scientific Inc.), *lane 2*—*A. longissima*, *lane 3*—*Ae. variabilis*, *lane 4*—*Ae. variabilis* × *S. cereale*, *lane 5*—*Ae. umbellulata*, *lane 6*—*S. cereale* ‘Strzękęcińskie,’ *lane 7* - triticale ‘Lamberto’; **b** leaf of (*Ae. variabilis* × *S. cereale*) × triticale infected by *B. graminis*; **c** no symptoms of *B. graminis* infection

During the vegetation period, the level of powdery mildew natural infection was evaluated according to COBORU (Cultivated Varieties National Research Centre) recommendations on a 9° scale, where 9 is the most favourable state for agriculture (Fig. 1b, c). The means of powdery mildew expression scores in BC_1_F_1_, BC_2_F_1_, BC_2_F_2_ hybrids, *Ae. variabilis* × *S. cereale* ampiploids and triticale ‘Lamberto’ were compared each year to the results of PCR amplification of *Pm13* marker using ANOVA calculations and Tukey’s HSD test.

## Results

### Pm13 marker analysis and powdery mildew reaction in parental forms

The amplification products of 517 bp in size were found in DNA extracts of *Ae. longissima* (PI 604112), *Ae. variabilis* and 20 plants of *Ae. variabilis* × *S. cereale*, which were used in further crosses with triticale. The bands of all samples gave clear and strong fluorescence after separation (Fig. 1a). The marker for *Pm 13* (517 bp) was not identified in rye ‘Strzekęcińskie’ (used for production of *Ae. variabilis* × *S. cereale* ampihiploids, Wojciechowska and Pudelska 1999) and triticale ‘Lamberto.’ The powdery mildew expression mean scores in *Ae. variabilis* were made in three subsequent years of experiments and ranged between 8.05 and 8.25 (Table 3). The observations of the infection symptoms conducted on triticale ‘Lamberto’ showed much lower tolerance to powdery mildew. The mean scores of infection ranged between 2.85 and 2.95 (Table 3).

### Identification of particular mitotic chromosomes of parental forms

**Fig. 2:**
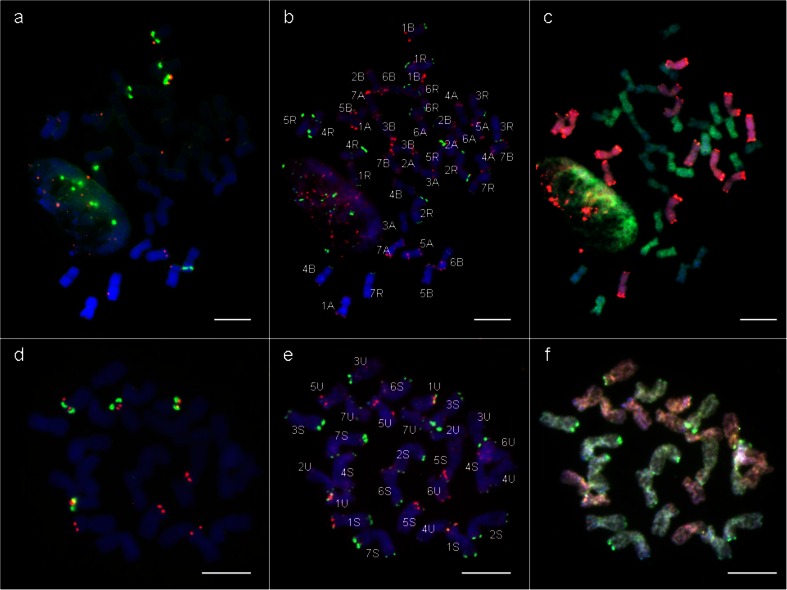
Fluorescence in situ hybridization (FISH) using 5S and 25S rDNA (**a**, **d**); pAs1 and pSc119.2 (**b**, **e**) repetitive DNA probes, and genomic in situ hybridization (GISH) on mitotic chromosomes of triticale (*× Triticosecale* Wittm.) ‘Lamberto’ (**a**, **b**, **c**) and *Ae. variabilis* Eig. (**d**, **e**, **f**). On the GISH images: **c** the R-genome is visualized in *red*, the A-genome in *green* and the B-genome in *blue*; **f** the U^v^-genome is visualized in *red* and the S^v^-genome in *green. Scale bar*s: 10 μm

**Fig. 3:**
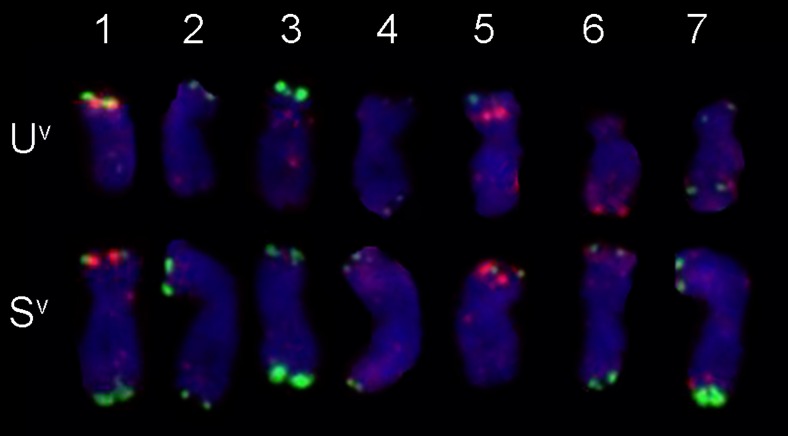
Representative karyotype of *Ae. variabilis* metaphase chromosomes after fluorescence in situ hybridization with signals originating pAs1 (*red*) and pSc119.2 (*green*)

The chromosome composition of *Ae. variabilis* (U^v^U^v^S^v^S^v^) and triticale ‘Lamberto’ (AABBRR), used as parental forms in presented distant crossing were studied (Fig. 2). The analysis were made using 5S rDNA, 25S rDNA (Fig. 2a, d), pSc119.2 and pAs1 probes (Fig. 2b, e) and multicolour GISH with total genomic DNA used as a probe (Fig. 2c, f). Identification of particular chromosomes of A- and B-genome, R-genome, U^u^-genome and S^l^-genome was made basing on previous reports of Cuadrado and Jouve (2002), Schneider et al. (2003, 2005) and Badaeva et al. 1996a, b and 2004, respectively and chromosome arms ratio. The rDNA-FISH experiment on chromosomes of triticale ‘Lamberto’ (2*n* = 6*x* = 42 chromosomes, AABBRR) resulted in 12 signals of 5S rDNA (on chromosomes 1A, 5A, 1B, 5B, 1R and 5R) and 6 signals of 25S rDNA (on chromosomes: 1B, 6B and 1R; Fig. 2a). By contrast, rDNA-FISH on *Ae. variabilis* (U^v^U^v^S^v^S^v^) chromosomes showed 8 signals of 5S rDNA in 1U^v^, 5U^v^, 1S^v^ and 5S^v^ chromosomes and 8 signals of 25S rDNA in 1U^v^, 5U^v^, 5S^v^ (weak) and 6S^v^ (weak) chromosomes (Fig. 2d). The same locations of rDNA signals appeared on chromosomes of *Ae. variabilis* × *S. cereale* amphiploid. The repetitive sequence FISH (seq-FISH) with pSc 119.2 and pAs1 probes resulted in specific patterns on chromosomes of triticale ‘Lamberto’ and *Ae. variabilis*. The chromosomes of A-genome of triticale carried only pAs1 signals, mainly on the distant and pericentromeric regions (Fig. 2b). The most distinguishable chromosome was 7A with strong pAs1 signal on the short arm. The pSc 119.2 and pAs1 signal locations on chromosomes of B-genome of triticale were more diversified and appeared also in interstitial regions. R-genome chromosomes of triticale had strong pSc119.2 sites and weak, dispersed pAs1 signals. The locations of pSc119.2 sites on 2R and 3R chromosomes were similar, but the difference of chromosome arms length allowed to distinguish those two. The chromosomes U^v^-genome of *Ae. variabilis* (Figs. 2e and 3) carried both the pSc119.2 sites and the pAs1 sites. The strongest pSc119.2 signal was observed in the telomeric region of 3U^v^ chromosome. The pAs1 sites were located both on distal and interstitial chromosomes. The most characteristic pattern was observed on 6U^v^ chromosome. The pSc119.2 and pAs1 probes hybridized also with S^v^-genome chromosomes (Fig. 3). The pSc119.2 sites were located on the telomeric regions of chromosomes with an exception of long arm of 5S^v^. The strongest signals were observed on the long arms of 3S^v^ and 7S^v^ chromosomes. The pAs1 sites were mostly dispersed. Distal regions of chromosome 4S^v^ and short arm of chromosome 7S^v^ carried the most visible signals of pAs1.

### Evaluation of crossing efficiency

106 flowers of triticale ‘Lamberto’ were pollinated by the pollen of *Ae. variabilis* × *S. cereale* forms (Table 1). 19 F_1_ seeds were obtained, that indicates 18 % of crossing efficiency (CE). Six F_1_ plants were germinated and evaluated using GISH analysis. Backcrossing of 68 flowers of F_1_ hybrids with the triticale ‘Lamberto’ pollen resulted in obtaining of 17 seeds of BC_1_F_1_ hybrid generation (CE = 25 %). Five BC_1_F_1_ plants were chosen on the basis of molecular marker (*Pm13*) test and cytogenetic analysis of mitotic chromosomes of root meristems for further crossing with triticale. After crossing of 330 flowers with triticale pollen, 25 seeds of BC_2_F_1_ generation were obtained. Thereafter, 15 plants were chosen for further hybridizations. 329 flowers of BC_2_F_1_ hybrids were self-pollinated, that resulted in 50 seeds of BC_2_F_2_ generation.

### Evaluation of introgression of *Ae. variabilis* chromatin in triticale hybrids

**Fig. 4:**
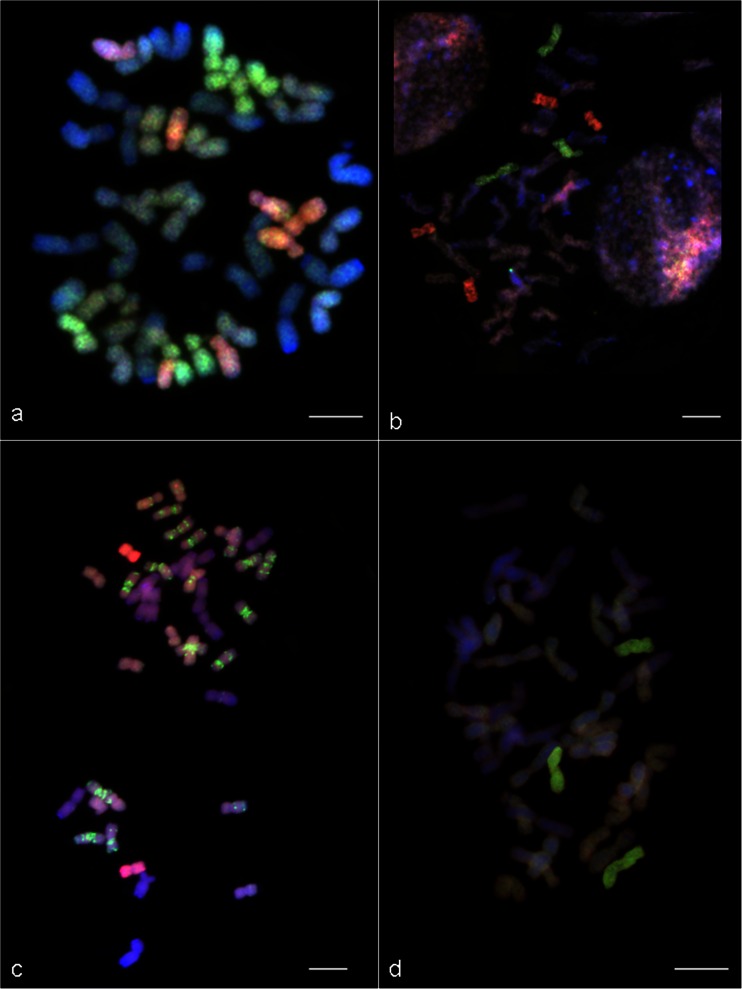
Genomic in situ hybridization (GISH) on mitotic chromosomes of (*Ae. variabilis* × *S. cereale*) × triticale ‘Lamberto’ hybrids. On the GISH images, the R-genome is visualized in *blue*, the A-genome and the B-genome in *grey*; the U^v^-genome is visualized in *red* and the S^v^-genome in *green*. **a** F_1_ hybrid with 14 chromosomes of *Ae. variabilis* (7 chromosomes of U^v^-genome and 7 chromosomes of S^v^-genome). **b** BC_1_F_1_ hybrid with 7 chromosomes of *Ae. variabilis* (4 chromosomes of U^v^-genome and 3 chromosomes of S^v^-genome). **c** BC_1_F_1_ hybrid with 2 chromosomes from U^v^-genome of *Ae. variabilis* and 21 chromosomes of triticale with introgression of S^v^-genome chromatin. **d** BC_2_F_1_ hybrid with 3 chromosomes from of S^v^-genome of *Ae. variabilis. Scale bars*: 10 μm

The correct establishing of the introgression of *Ae. variabilis* chromatin carrying the resistance to powdery mildew was assured by combining the GISH and FISH methods with molecular marker (*Pm13*) analysis and the results of infection scoring. The chromosome constitution of six F_1_ (*Ae. variabilis* × *S. cereale*) × triticale hybrids consist of 28 chromosomes of triticale (14 chromosomes of A- and B-genomes and 14 R-genome chromosomes), seven U^v^-genome chromosomes and seven S^v^-genome chromosomes, which were detected by probing with U^u^- and S^l^-genomic DNA and blocking with total DNA of triticale (AABBRR) (Table 2, Fig. 4a). FISH experiment with 4 kinds of probes allowed to distinguish chromosomes from each group (group-1 to group-7).

**Table 2:** Cytogenetic analysis of F_1_ to BC_2_F_2_ hybrids of triticale ‘Lamberto’ × (*Ae. variabilis* × *S. cereale*) carrying *Ae. variabilis* chromatin with *Pm13* marker

Generation	Number of plants	Chromosome composition	Total number of chromosomes
F_1_	6	14″ + 1′1U^v^ + 1′2U^v^ + 1′3U^v^ + 1′4U^v^ + 1′5U^v^ + 1′6U^v^ + 1′7U^v^ + 1′1S^v^ + 1′2S^v^ + 1′3S^v^ + 1′4S^v^ + 1′5S^v^ + 1′6S^v^ + 1′7S^v^	42
BC_1_F_1_	1	16″ + 1′3B + 1′2U^v^ + 1′3U^v^ + 1′4U^v^ + 1′6U^v^ + 1′2S^v^ + 1′3S^v^ + 1′4S^v^	40
	1	16″ + 1′3U^v^ + 1′4U^v^ + 1′2S^v^ + 1′3S^v^ + 1′4S^v^	37
	1	17″ + 1′2B + 1′2U^v^ + 1′3U^v^ + 1′4U^v^ + 1′2S^v^ + 1′3S^v^ + 1′4S^v^	41
	1	17″ + 1′2U^v^ + 1′3U^v^ + 1′4U^v^ + 1′6U^v^ + 1′7U^v^ + 1′2S^v^ + 1′3S^v^ + 1′4S^v^ + 1′7S^v^	43
	1	17″ + 1′2U^v^ + 1′3U^v^ + 1′4U^v^ + 1′6U^v^ + 1′2S^v^ + 1′3S^v^ + 1^v^4S^v^ + 1′7S^v^	42
BC_2_F_1_	3	20″ + 1′3S^v^/3B	41
	4	21″ + 1′3S′	43
	6	20″ + 1″3S^v^/3B	42
	1	20″ + 1′3S^v^/3B + 1′2S^v^	43
	1	20″ + 1′2B + 1″3S^v^/3B + 1′2S^v^	44
BC_2_F_2_	9	21″ + 1′3S^v^	43
	10	20″ + 1″3S^v^/3B	42
	7	21″ + 1″3S^v^	44

xx″- number of pairs of triticale chromosomes, 1″xy- one pair of y-genome chromosomes of group-x; 1′xy- a singular group-x chromosome of y-genome; 1″xy/xz- substitution pair of chromosomes. The nomenclature and abbreviation of the genetic stocks of hybrids were described according Raupp et al. 1995 (http://wheat.pw.usda.gov/ggpages/nomenclature.html)

Afterwards, five of 17 plants of the BC_1_F_1_ generation carried *Pm13* marker, which was correlated with the infection scores that ranged from 6 to 8, whereas the another 12 plants were more infected, which was comparable with the infection level of triticale ‘Lamberto’ (Table 3). In those 5 hybrids (with *Pm13* marker) the total number of chromosomes varied from 37 to 43 (Table 2). The number of U^v^-genome chromosomes was between 2 and 5, the number of S^v^ chromosomes was 3–4, the number of R-genome chromosomes was 14 in each plant and the A and B-genome chromosomes number varied from 18 to 21 (Fig. 4b). The 12 other plants, without *Pm13* marker, had large number of intergeneric translocations. The GISH analysis showed the chromosomes of A- and B-genome with the translocations of S-genome chromosome segments (Fig. 4c). Selected five BC_1_F_1_ hybrids (with *Pm13* marker) were backcrossed with triticale pollen. The molecular analysis showed that the 3 of 5 BC_1_F_1_ plants reproduced 15 descendants (BC_2_F_1_) with the *Pm13* marker (Table 1). The infection scores of those group of hybrids were significantly different in comparison with hybrids without *Pm13* marker and triticale ‘Lamberto.’

**Table 3:** Evaluation of the natural infection level caused by *B. graminis* on the BC_1_F_1_, BC_2_F_1_ and BC_2_F_2_ hybrids of (*Ae. variabilis* × *S. cereale*) × triticale ‘Lamberto’ that carried or did not carry the *Pm13* marker

Generation	Number of plants				Means (range) of infection scores			
	With *Pm13* marker		Without *Pm13* marker		With *Pm13* marker		Without *Pm13* marker	
					1	2	3	4
	*Ae. variabilis*	hybrids	triticale ‘Lamberto’	hybrids	*Ae. variabilis*	hybrids	triticale ‘Lamberto’	hybrids
BC_1_F_1_	20	5	20	12	8.25 (7–9)	7.40 (6–8)	2.90 (2–4)	3.50 (2–4)
BC_2_F_1_	20	15	20	10	8.10 (7–9)	6.80 (6–8)	2.95 (2–4)	2.90 (2–4)
BC_2_F_2_	20	26	20	24	8.05 (7–9)	6.62 (6–8)	2.85 (2–4)	2.92 (2–4)
Tukey’s Honest Significant Difference (HSD) test								
Generation	HSD level		1 vs 2	1 vs 3	1 vs 4	2 vs 3	2 vs 4	3 vs 4
	HSD_0.05_	HSD_0.01_						
BC_1_F_1_	0.81	1.00	*P* < 0.05	*P* < 0.01	*P* < 0.01	*P* < 0.01	*P* < 0.01	n/s
BC_2_F_1_	0.68	0.83	*P* < 0.01	*P* < 0.01	*P* < 0.01	*P* < 0.01	*P* < 0.01	n/s
*BC*_*2*_ *F*_*2*_	0.59	0.72	*P* < 0.01	*P* < 0.01	*P* < 0.01	*P* < 0.01	*P* < 0.01	n/s

The U^v^-genome chromosomes were not identified in all of 15 plants of BC_2_F_1_ generation, but 1 to 3 chromosomes of S^v^ -genome appeared in those plants (Fig. 4d). FISH analysis showed that 3 plants carried 41 chromosomes with one chromosome 3S^v^ and the lack of 3B chromosome pair. Another 4 plants possessed additional chromosome 3S^v^. The 6 other plants carried substitution pair of 3S^v^/3B chromosomes. Moreover, one of BC_2_F_1_ hybrids had a substitution pair of 3S^v^/3B chromosomes and one additional chromosome 2S^v^. The other singular plant carried: a substitution pair of 3S^v^/3B chromosomes, an one additional 2S^v^ chromosome and one chromosome 2B (Table 2).

In the BC_2_F_2_ generation the S^v^ -genome chromosomes were eliminated in 24 plants, however in 26 hybrids 1–2 chromosomes of S^v^ -genome were identified and the range of triticale chromosomes was the same as in the previous generation. FISH experiments allowed to distinguish 9 plants with one, additional chromosome 3S^v^, 10 plants with a substitution pair of 3S^v^/3B chromosomes and 7 plants with an additional pair of 3S^v^ chromosomes. *Pm13* marker was identified only in plants with introgression of *Aegilops* chromatin, which was correlated with the powdery mildew infection scores (Table 3).

### Chromosome pairing behaviour in BC_2_F_1_ and BC_2_F_2_ of (*Ae. variabilis* × *S. cereale*) × triticale hybrids

**Fig. 5:**
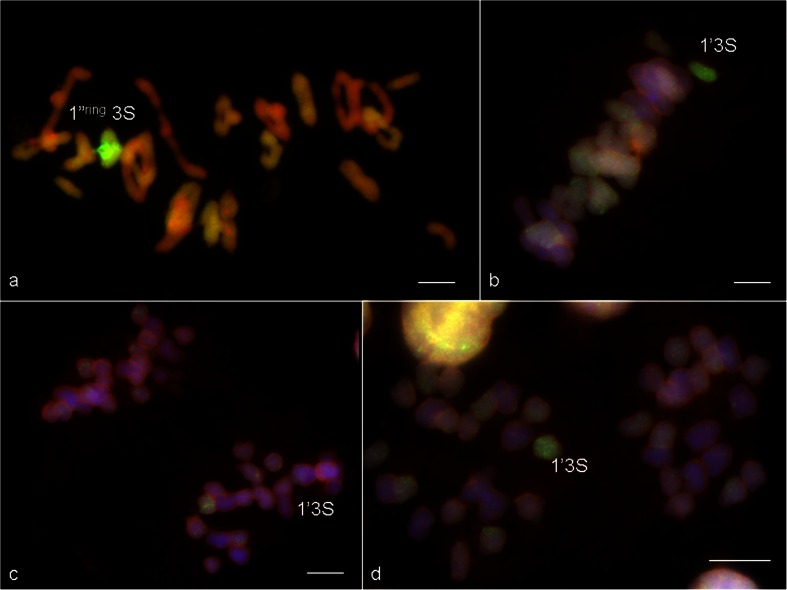
Chromosome associations at meiosis of pollen mother cells of BC_2_F_2_ (*Ae. variabilis* × *S. cereale*) × triticale ‘Lamberto’ hybrids. GISH images created using S^v^-genome genomic DNA as a probe (*green*), with blocking genomic DNA of triticale. Chromosomes were counterstained with propidium iodide (**a**) or DAPI (**b**, **c**, **d**). **a** One 3S^v^/3S^v^ bivalent in 3S^v^/3B substitution line (2*n* = 42) at metaphase I of meiosis. **b** One 3S^v^ univalent in 3S^v^ addition line (2*n* = 43) at **b** metaphase I, **c** anaphase I and **d** telophase I of meiosis. *Scale bars*: 10 μm

The multicolour GISH allowed to distinguish the S^v^-genome chromosomes (green) and the triticale chromosomes (Fig. 5a–d). Chromosome configuration means at MI of meiosis in PMCs were examined in selected hybrid plants of BC_2_F_1_ with total number of chromosomes amounting 42, that carried a substitution pair of 3S^v^/3B chromosomes (Table 4) and in BC_2_F_2_ hybrids divided in two groups. First group consisted of plants with 42 chromosomes, having a substitution pair of 3S^v^/3B chromosomes (Table 5), while second group associated the plants with 43 chromosomes having an additional 3S^v^ chromosome (Table 6).

**Table 4:** Analysis of chromosome configurations during metaphase I of meiosis of PMCs of five BC_2_F_1_ hybrids (2*n* = 42) with an introgression of a 3S^v^ chromosome pair of *Ae. variabilis*

Plant number (number of chromosomes)	Number of PMC’s	Mean and range of chromosome configurations at metaphase I												
		*Bivalents*									*Univalents*			
		Rods				Rings				∑	S	AB	R	∑
		∑	AB/AB	R/R	S/S	∑	AB/AB	R/R	S/S					
4/3 (42)	10	12.1 (8–17)	6.8 (4–10)	5 (4–6)	0.3 (0–1)	5.2 (1–9)	3.4 (0–6)	1.5 (0–3)	0.3 (0–1)	17.3 (9–20)	0.8 (0–2)	5.4 (2–16)	1.2 (0–6)	7.4 (2-24)
4/5 (42)	10	11.6 (10–14)	6.8 (5–10)	4.3 (3–5)	0.5 (0–1)	6.5 (2–9)	3.8 (0–6)	2.2 (1–4)	0.5 (0–1)	18.1 (16–20)	0	4.8 (2–8)	1 (0–4)	5.8 (2-10)
4/6 (42)	10	12.3 (9–15)	7.5 (4–10)	4.6 (3–6)	0.2 (0–1)	5.9 (3–8)	3.1 (0–6)	2.4 (1–4)	0.4 (0–1)	18.2 (15–20)	1 (0–2)	4.4 (0–10)	0.2 (0–2)	5.6 (2-12)
4/10 (42)	10	11.2 (5–15)	6.4 (3–9)	4.6 (2–7)	0.2 (0–1)	6.7 (2–9)	4.2 (0–7)	2.2 (0–5)	0.3 (0–1)	17.9 (14–20)	1 (0–2)	4.8 (0–12)	0.4 (0–2)	6.2 (2-14)
4/11 (42)	10	13.9 (10–17)	8.2 (6–12)	5.4 (4–7)	0.3 (0–1)	4.7 (1–9)	2.8 (0–6)	1.5 (0–3)	0.4 (0–1)	18.6 (16–20)	0.8 (0–2)	3.8 (2–8)	0.2 (0–2)	4.8 (2-10)
Mean		12.22 (5-17)	7.14 (3–12)	4.78 (2–7)	0.30 (0–1)	5.80 (1–9)	3.46 (0–7)	1.96 (0–5)	0.38 (0–1)	18.02 (9–20)	0.72 (0–2)	4.64 (0–16)	0.60 (0–6)	5.96 (2–24)
ANOVA	F	2.05	1.66	1.66	0.68	1.21	0.81	1.36	0.27	0.54	1.97	0.35	1.52	0.54
*summary*	*P*	0.103368	0.175949	0.175949	0.609437	0.319804	0.525409	0.262852	0.895752	0.707101	0.115345	0.842649	0.212455	0.707101

**Table 5:** Analysis of chromosome configurations during metaphase I of meiosis of PMCs of five BC_2_F_2_ hybrids (2*n* = 42) with an introgression of a 3S^v^ chromosome pair of *Ae. variabilis*

Plant number (number of chromosomes)	Number of PMC’s	Mean and range of chromosome configurations at metaphase I												
		*Bivalents*									*Univalents*			
		Rods				Rings				∑	S	AB	R	∑
		∑	AB/AB	R/R	S/S	∑	AB/AB	R/R	S/S					
4/6/1 (42)	10	12.4 (10–15)	7.5 (6–9)	4.7 (4–6)	0.2 (0–1)	6.8 (4–9)	4.2 (2–6)	2.1 (1–3)	0.5 (0–1)	19.2 (18–20)	0.6 (0–2)	2.6 (0–4)	0.4 (0–2)	3.6 (2–6)
4/6/3 (42)	10	12.2 (10–15)	7.4 (6–10)	4.7 (4–6)	0.1 (0–1)	6.9 (4–9)	4.0 (2–6)	2.2 (1–3)	0.7 (0–1)	19.1 (17–20)	0.6 (0–2)	3.0 (0–6)	0.2 (0–2)	3.8 (2–8)
4/10/5 (42)	10	12.0 (9–15)	7.1 (5–9)	4.8 (4–7)	0.1 (0–1)	6.8 (2–9)	4.3 (0–7)	1.9 (0–3)	0.6 (0–1)	18.8 (16–21)	0.6 (0–2)	3.2 (0–8)	0.6 (0–4)	4.4 (0–10)
4/10/7 (42)	10	14.0 (10–17)	8.2 (6–12)	5.4 (4–7)	0.4 (0–1)	4.5 (1–9)	2.8 (0–6)	1.5 (0–3)	0.2 (0–1)	18.5 (16–20)	0.8 (0–2)	4.0 (2–8)	0.2 (0–2)	5.0 (2–10)
4/10/8 (42)	10	12.2 (8–17)	6.9 (4–10)	5.0 (4–6)	0.3 (0–1)	5.1 (1–9)	3.9 (0–6)	1.5 (0–3)	0.3 (0–1)	17.3 (9–20)	0.8 (0–2)	5.4 (2–16)	1.2 (0–6)	6.2 (2–12)
mean		12.56 (8–17)	7.42 (4–12)	4.92 (4–6)	0.22 (0–1)	6.14 (1–9)	3.84 (0–7)	1.84 (0–3)	0.46 (0–1)	18.7 (9–20)	0.68 (0–2)	3.40 (0–16)	0.52 (0–6)	4.60 (0–12)
ANOVA	F	1.67	1.05	0.97	0.97	2.21	1.13	1	1.81	1.56	0.12	1.13	1.22	1.56
*summary*	*P*	0.173585	0.392246	0.433323	0.433323	0.082983	0.354443	0.417531	0.143525	0.201351	0.974664	0.354443	0.315690	0.201351

**Table 6:** Analysis of chromosome configurations during metaphase I of meiosis of PMCs of four BC_2_F_2_ hybrids (2*n* = 43) with additional 3S^v^ chromosome of *Ae. variabilis*

Plant number (number of chromosomes)	Number of PMC’s	Mean and range of chromosome configurations at metaphase I												
		*Bivalents*									*Univalents*			
		Rods				Rings				∑	S	AB	R	∑
		∑	AB/AB	R/R	S/S	∑	AB/AB	R/R	S/S					
4/6/2 (43)	10	12.2 (10–15)	7.4 (6–9)	4.8 (4–6)	0	6.3 (3–8)	4.3 (2–6)	2 (1–3)	0	18.5 (17–20)	1	4.6 (2–6)	0.4 (0–2)	6 (3–9)
4/10/2 (43)	10	8.6 (7–10)	5.3 (4–6)	3.3 (2–5)	0	11.1 (9–14)	7.6 (6–9)	3.5 (1–5)	0	19.7 (19–21)	1	2.2 (0–4)	0.4 (0–2)	3.6 (1–5)
4/10/3 (43)	10	9.2 (7–11)	6.1 (4–8)	3.1 (1–5)	0	9.9 (6–13)	6.1 (2–8)	3.8 (1–6)	0	19.1 (17–21)	1	3.6 (0–8)	0.2 (0–2)	4.8 (1–9)
4/10/4 (43)	10	9.6 (7–13)	6.7 (6–8)	2.9 (1–5)	0	9.8 (7–13)	6.5 (5–10)	3.3 (2–4)	0	19.4 (18–21)	1	2.2 (0–4)	1 (0–4)	4.2 (1–7)
Mean		9.9 (7–15)	6.38 (4–9)	3.53 (1–6)	0	9.28 (3–14)	6.13 (2–10)	3.15 (1–6)	0	19.18 (17–21)	1	3.15 (0–8)	0.5 (0–4)	4.65 (1–9)
ANOVA	F	12.26	8.07	5.45	0	14.34	8.93	4.91	0	2.49	0	4.48	1.26	2.49
Summary	*P*	<0.0001	0.000307	0.003408	1	<0.0001	0.000148	0.005818	1	0.075836	1	0.008997	0.302638	0.075836
Tukey’s HSD test	HSD_0.05_	1.73	1.2	1.41	n/a	2.08	1.75	1.37	n/a	n/a	n/a	2.11	n/a	n/a
	HSD_0.01_	2.14	1.49	1.75	n/a	2.59	2.17	1.69	n/a	n/a	n/a	2.62	n/a	n/a
	4/6/2 vs 4/10/2	*P* < 0.05	*P* < 0.01	*P* < 0.05	n/a	*P* < 0.01	*P* < 0.01	*P* < 0.05	n/a	n/a	n/a	*P* < 0.05	n/a	n/a
	4/6/2 vs 4/10/3	*P* < 0.05	*P* < 0.05	*P* < 0.05	n/a	*P* < 0.01	*P* < 0.05	*P* < 0.05	n/a	n/a	n/a	n/s	n/a	n/a
	4/6/2 vs 4/10/4	*P* < 0.05	n/s	*P* < 0.01	n/a	*P* < 0.01	*P* < 0.01	n/s	n/a	n/a	n/a	*P* < 0.05	n/a	n/a
	4/10/2 vs 4/10/3	n/s	n/s	n/s	n/a	n/s	n/s	n/s	n/a	n/a	n/a	n/s	n/a	n/a
	4/10/2 vs 4/10/4	n/s	*P* < 0.05	n/s	n/a	n/s	n/s	n/s	n/a	n/a	n/a	n/s	n/a	n/a
	4/10/3 vs 4/10/4	n/s	n/s	n/s	n/a	n/s	n/s	n/s	n/a	n/a	n/a	n/s	n/a	n/a

The variance analysis of the chromosome configurations in BC_2_F_1_ plants with 42 chromosomes, that carried a substitution pair of 3S^v^/3B chromosomes showed that the differences between the means of chromosome configurations were not significant (Table 4). The mean of total number of bivalents were 18.02. Bivalents ranged from 9 to 20 per cell. The mean of rod bivalents was nearly two times higher than the mean of ring bivalents (12.22; 5.80; respectively). Similarly, the mean of rod bivalents of A-, B- and R-genome was considerably higher than ring bivalents of those genomes. Considering the S^v^-genome bivalents, the mean number of S^v^/S^v^ rod bivalents and S^v^/S^v^ ring bivalents was almost equal (0.30 and 0.38, respectively). The mean of S^v^-genome univalents was 0.72 and the number of univalents ranged between 0 and 2. The mean chromosome configuration for five analysed plants (2*n* = 42 chromosomes) with a substitution pair of 3S^v^/3B chromosomes was 5.96 I + 18.02 II (12.22 rod + 5.80 ring).

The ANOVA test for BC_2_F_2_ hybrids with the same chromosome constitution (20′″ + 3S^v^′) obtained from different BC_1_F_1_ plants, carrying a substitution pair of 3S^v^/3B chromosomes showed that the differences between means of the chromosome configurations of particular hybrids were not statistically significant. The mean (and the range) of bivalents per PMC was 18.7 (9–20) and was similar to the results in BC_2_F_1_ hybrids. The same situation appeared considering the means of rod bivalents, ring bivalents and univalents, where mean chromosome configuration for five analysed BC_2_F_2_ plants (2*n* = 42 chromosomes) with a substitution pair of 3S^v^/3B chromosomes was 4.60 I + 18.70 II (12.56 rod + 6.14 ring). The mean of rod and ring S^v^-genome bivalents was approximate (0.22 and 0.46; respectively). The comparison of ANOVA results of chromosome configuration between BC_2_F_1_ and respective BC_2_F_2_ progeny hybrids shows that the differences in means are not significant. Considering the S^v^-genome univalents, the mean in BC_2_F_2_ plants (Table 5) was lower than in BC_2_F_1_ plants (Table 4). Five of six hybrids of BC_2_F_1_ (42 chromosomes each), which carried a substitution pair of 3S^v^/3B chromosomes were evaluated (Table 2). All of them were the progeny of the most fertile hybrid line no. 4 (Table 1).

Chromosome configuration means at MI of meiosis in PMCs were also examined in four BC_2_F_2_ hybrid plants (2*n* = 43 chromosomes) carrying additional chromosome 3S^v^. The mean chromosome configuration for this group was 4.65 I + 19.18 II (9.9 rod + 9.28 ring). The ANOVA and Tukey’s HSD test showed that the differences of chromosomes configuration means between plants with the same chromosome constitution (21′″ + 3S^v^′) obtained from different BC_2_F_1_ plants (4/6 and 4/10) were significant. The differences affected the means of A-genome, B-genome and R-genome rod and ring bivalents and also means of univalents of A- and B-genome (Table 6).

## Discussion

Considering the growing tendency in brakeage of triticale resistance to fungal diseases, especially powdery mildew, and from the other hand, the narrow genetic diversity of triticale could lead to the conclusion that it is necessary to utilize the wild Triticeae relatives to enrich the genetic pool of cultivated triticale. The gene order in Poaceae species is generally conserved (Chantret et al. 2008) and the synteny facilitates comparative genomics analyses in grass families (Abrouk et al. 2010). Therefore, it could be expected that the region of chromosome 3S^l^ of *A. longissima* that is responsible for powdery mildew resistance could be collinear with the same region in the chromosome 3S^v^ of *Ae. variabilis* (2*n* = 4*x* = 28, U^v^U^v^S^v^S^v^). Nonetheless, there are discrepant reports concerning the powdery mildew resistance of *Ae. variabilis*. From the one side, Spetsov and Iliev (1991) obtained a disomic addition line (2*n* = 44) by crossing wheat cv. ‘Roussallka’ with *Ae. variabilis*, that manifested a high powdery mildew resistance in seedling and in adult plant stage. From the other side, Cenci et al. (2003) reported that disomic line of wheat cv. ‘Chinese Spring’ 3S^v^ (K-2) and the derived ditelosomic 3S^v^S (K-2/S*v*S) addition lines from *Ae. variabilis* (Yang et al. 1996) were susceptible, with strong powdery mildew symptoms and abundant sporulation. However, the assumption of a possible synteny between the S-genome chromosomes became meaningful, considering the verification of available powdery mildew STS markers made by Stępień et al. (2001), which showed that *Pm13* marker was present in *Ae. speltoides* (accessions 2056, 2067, d10, d42, d50) that also carry S-genome chromosomes. In presented study, the *Ae. variabilis* and the *Ae. variabilis* × *S. cereale* amphiploids carrying *Pm13* marker manifested a low powdery mildew reaction, confirmed by infection scores made on 20 plants each year of the experiment (Fig. 1c; Table 3). In comparison, triticale ‘Lamberto’ was much more infected, which was confirmed by Tukey’s HSD test (Fig. 1c; Table 3). Moreover, 1402 Polish isolates of *B. graminis* are reported to be 100 % virulent to triticale ‘Lamberto’ in three subsequent years of experiment (2008–2010) carried out by Czembor et al. (2014). Furthermore, the molecular analysis showed the *Pm13* marker was not present in triticale ‘Lamberto’ (Table 3). The *Pm13* marker is located on the distal region of the short arm of chromosome 3S^l^ (Cenci et al. 2003). In purpose to identify the particular chromosomes of *Ae. variabilis*, the FISH experiment with repetitive sequences as probes was carried out. The location of 25S rDNA and 5S rDNA signals in U- and S-genome chromosomes of *Ae. variabilis* were similar like in the ancestor species, considering chromosomes 1U^u^ 5U^u^ and 5S^l^ and 6S^l^ of *Ae. umbellulata* and *Ae. longissima*, respectively (Badaeva et al. 1996b). However, the 25S rDNA signals on 1S^l^, 3S^l^ and 6U^u^ chromosomes were not present on the homologue chromosomes of *Ae. variabilis*. There were also some differences in pSc119.2 signals pattern between diploid ancestors (Badaeva et al. 1996a) and *Ae. variabilis* (Fig. 3). There were no signals in the telomeric regions of long arms of 2U^v^, 3U^v^, 5U^v^ and 6U^v^ chromosomes. When comparing pAs1 signals on the U-genome chromosomes, small, dispersed signals were observed on 1U^v^, 3U^v^ and 5U^v^ chromosomes. Moreover, Badaeva et al. (1996a) did not observed the pAs1 signals on S-genome chromosomes of *Ae. longissima*, however chromosomes of *Ae. variabilis* carried weak, scattered landmarks on both arm of each chromosome and strong site on distal region of long arm of 7S^v^ chromosome. The cytogenetic analysis of triticale ‘Lamberto’ chromosomes revealed also some novel data. The elimination of 25-5.8-18S rDNA was observed in 1A chromosome of triticale, comparing to 1A of wheat. The rDNA aberrations are probably connected with the changes in ploidy level, which commonly appear in hybrids (Shcherban et al. 2008).

Knowing the cytogenetic markers distribution on the chromosomes of parental forms (*Ae. variabilis* × *S. cereale* amphiploids and triticale ‘Lamberto’), and the results of *Pm13* molecular marker analysis connected with the evaluation of natural infection by *B. graminis*, the study of hybrid generations of (*Ae. variabilis* × *S. cereale*) × triticale ‘Lamberto’ were made. As expected, the F_1_ hybrids (2*n* = 6*x* = 42, U^v^S^v^ABRR) carried 7 chromosomes of U^v^-, S^v^-, A- and B-genome and complete set of 14 chromosomes of R-genome. The chromosome composition of F_1_ hybrids was anticipated on the basis of related studies, i.e. in the study of *Aegilops biuncialis* (2*n* = 4*x* = 28, UUMM) × wheat (2*n* = 6*x* = 42, AABBDD) hybridizations (Schneider et al. 2005), the chromosome set of F_1_ hybrids were parallel (ABDUM, 2*n* = 5*x* = 35), with only one difference, that in case of (*Ae. variabilis* × *S. cereale*) × triticale hybridizations, R-genome chromosomes can pair and behave in diploid manner. The crossing of F_1_ hybrids with triticale pollen had an influence on reduction of the *Aegilops* chromosomes in one group of BC_1_F_1_ plants and appearing of S^v^/AB translocations in the latter group of BC_1_F_1_ plants. Marker analysis showed that plants with *Aegilops* chromosomes carried also *Pm13* marker. Moreover, those plants were much more tolerant for *B. graminis* infection (Table 3). The further backcrossing of selected BC_1_F_1_ hybrids with triticale pollen resulted in elimination of *Aegilops* chromosomes. There was lack of *Aegilops* chromatin in 9 BC_2_F_1_ plants. On the other hand, FISH/GISH analysis allowed to distinguish chromosome(s) 3S^v^ in each of 15 BC_2_F_1_ plants and in addition, one chromosome 2^v^ in 2 plants, where also *Pm13* marker was identified. Moreover, the intensity of the level of powdery mildew infection on those plants was lower, when comparing with triticale ‘Lamberto’ and hybrids without *Pm13* marker. Two subsequent backcrosses resulted in the elimination of unneeded *Aegilops* chromosomes and allow to select the plants with the S-genome chromosomes carrying the resistance. Therefore, the self-fertilization of BC_2_F_1_ was carried out to maintain the S-genome chromosome in BC_2_F_2_ hybrids. 26 of 50 hybrids had singular or a pair of 3S^v^ chromosomes, that carried *Pm13* marker and were more tolerant for *B. graminis* infection. It cannot be omitted, that the HSD test of the means of infection scores of hybrids with *Pm13* marker compared with the mean of infection scores of amphiploids (*Ae. variabilis* × *S. cereale*) shows the significant differences (Table 3), that points the tolerance for powdery mildew is a little bit lower in hybrids than in amphiploids, however is much higher than in triticale ‘Lamberto’ and hybrids without *Pm13* marker. It can be supposed that triticale ‘Lamberto’ carry a virulence factors, that have an influence on *Pm13* gene expression. Notwithstanding, the tolerance for powdery mildew was markedly improved in hybrids with *Pm13* marker. Afterwards, the genomic in situ hybridization was employed to study the 3S^v^ chromosome(s) behaviour in PMC’s of selected BC_2_F_1_ and BC_2_F_2_ hybrids of (*Ae. variabilis* × *S. cereale*) × triticale ‘Lamberto.’ There were no intergenomic chromosome configurations observed in the plant carrying 2S^v^ and/or 3S^v^ chromosomes, which is opposite to other published studies concerning intergenomic hybridizations between cultivated cereals and *Aegilops* species. For example, Molnár and Molnár-Láng (2010) reported the intergenomic rod and ring bivalents and trivalent between 2 M, 3 M, 3U and 7 M chromosomes of *Ae. biuncialis* and wheat (Chinese Spring *ph1b*) chromosomes. It is assumed, that triticale has the same controlling system of homologue chromosome pairing as wheat, that hampers the pairing of the chromosomes from different genomes. In wheat, homoeologous chromosome pairing and consequent recombination is suppressed by the function of the *Ph1* locus, localized on the long arm of chromosome 5B (Riley and Chapman 1958). The Chinese Spring *ph1b* (*CSph1b*) mutant genotype (Sears 1977), which lacks the *Ph1* locus, has been successfully used for the introgression of alien genetic material into the wheat genome by the induction of homoeologous pairing (Lukaszewski 2000). From this reason the intergenomic bivalent and trivalent appeared in Molnár and Molnár-Láng (2010) study. Considering presented study, FISH experiments showed that the pair of chromosomes 5B was present in all hybrids of each generation and probably is responsible for diploid-like pairing of chromosomes during meiosis, which was confirmed by ANOVA tests (Tables 4, 5 and 6) that demonstrated no differences in means of chromosome configurations between hybrid plants. However, Tukey’s HSD test showed the differences in means of bivalent configurations between BC_2_F_2_ progeny obtained from 4/6 plant compared with the progeny of 4/10 hybrid (Table 6). It can be supposed that S-genome chromatin has no influence on chromosome pairing of triticale chromosomes. In other words, the way of triticale chromosomes behaviour during first metaphase of meiosis of PMCs seems to be individual regarding to parental form. Furthermore, the way of 3S^v^ chromosome pairing and transmission to next generation is independent, diploid-like.

In conclusion, our study showed that molecular cytogenetics and marker-assisted selection combined with evaluation of powdery mildew infection constitute a useful tool for the resistance breeding. Using these methods we have obtained 26 plants carrying 3S^v^ chromosome(s) with the powdery mildew resistance, which can be used in the triticale breeding programmes. On the other hand, these genetic stocks could be used for sequencing the specific region of 3S^v^ chromosome, responsible for powdery mildew tolerance and for comparative studies with the *Pm13* gene sequence originated from *Ae. longissima*.
